# Safety envelope of pedestrians upon motor vehicle conflicts identified via active avoidance behaviour

**DOI:** 10.1038/s41598-021-82331-z

**Published:** 2021-02-17

**Authors:** Bingbing Nie, Quan Li, Shun Gan, Bobin Xing, Yuan Huang, Shengbo Eben Li

**Affiliations:** State Key Laboratory of Automotive Safety and Energy, School of Vehicle and Mobility, Tsinghua University, Beijing, 100084 China

**Keywords:** Risk factors, Engineering, Mechanical engineering

## Abstract

Human reaction plays a key role in improved protection upon emergent traffic situations with motor vehicles. Understanding the underlying behaviour mechanisms can combine active sensing system on feature caption and passive devices on injury mitigation for automated vehicles. The study aims to identify the distance-based safety boundary (“safety envelope”) of vehicle–pedestrian conflicts via pedestrian active avoidance behaviour recorded in well-controlled, immersive virtual reality-based emergent traffic scenarios. Via physiological signal measurement and kinematics reconstruction of the complete sequence, we discovered the general perception-decision-action mechanisms under given external stimulus, and the resultant certain level of natural harm-avoidance action. Using vision as the main information source, 70% pedestrians managed to avoid the collision by adapting walking speeds and directions, consuming overall less “decision” time (0.17–0.24 s vs. 0.41 s) than the collision cases, after that, pedestrians need enough “execution” time (1.52–1.84 s) to take avoidance action. Safety envelopes were generated by combining the simultaneous interactions between the pedestrian and the vehicle. The present investigation on emergent reaction dynamics clears a way for realistic modelling of biomechanical behaviour, and preliminarily demonstrates the feasibility of incorporating in vivo pedestrian behaviour into engineering design which can facilitate improved, interactive on-board devices towards global optimal safety.

## Introduction

Road traffic accident remains a worldwide public health concern and results in 1.3 million fatalities and 50 million injuries annually. As vulnerable road users (VRUs), pedestrians are exposed to high injury risks from collisions upon traffic collisions with motor vehicles on road^1^. Pedestrian reaction upon emergent traffic situations largely influences the occurrence and severities of accidents^2^. Multiple influencing factors on pedestrian collision risk shall be considered for identifying relatively the distance-based safety boundary of pedestrian and vehicles, including both human factors (such as kinematics, posture, gait, age^3–5^) and vehicle factors (such as impact velocity, front-end structural design, relative location^6–8^). Epidemiological studies have provided the mostly on-hand information on field while are in lack of precise and comprehensive description of human reactions right before the event. A series of experiments, simulations and analysis using physical and computational human surrogates, e.g., crash dummies, human body models^9–11^, have been devoted to identifying the passive influencing factors on pedestrian safety^12^. Yet, little work has been performed to quantify the natural behaviour of pedestrians upon traffic emergency. One major difficulty that hinders a systematic investigation is the precise extraction of pedestrian active avoidance behaviour in a well-controlled and near-real environment.

Pedestrian is one key element in the whole human–vehicle–road loop in road traffic scenarios. The uncertainties of human behaviour upon emergent traffic situations have long been a blind spot^13,14^, posing high challenges in safety system design. Upon traffic conflicts, pedestrians used vision as the main source of information to make a decision and control their motion^15,16^, and exhibited a natural “perception-decision-execution” ability in avoiding danger to a certain level. Such capability largely relies on their own “detection” capability (e.g., via vision information) of upcoming hazards. A quantitative description of pedestrian active behaviour is particularly necessary with the growing anticipation of highly automated vehicles (HAVs)^17^ and the resultant unexpected fatalities on road^18^ (e.g., the first pedestrian fatality caused by a tested automated vehicle, Arizona, USA, 2018). Existing safety-focused strategies for pedestrians are mostly focusing on the available motion information to predict subject trajectories, project future traffic interactions and compute time-to-collision (TTC). The use of TTC-related information includes a collision risk assessment metric and a cue for decision-making to start braking and/or steering^19^. Advancements in sensing technology on vehicles have been well capable of collecting pre-collision pedestrian information, making it possible to take pedestrian behaviour into account for deploying advanced safety devices.

Despite the influence of human factors on the safety subsequence, relevant regulations and standards have long been evaluating pedestrian protection performance using well-defined passive setup (i.e., purely lateral vehicle–pedestrian impact configuration^20,21^). Such a fact has been limiting the vehicle safety development to a very narrow sample size from the diverse real-world scenarios^13^. Active response of road users, including diverse posture and whole-body kinematics, proved to significantly influence the subsequent injury risks in collisions as evidenced by epidemiological and computational data^22,23^. In recent perspectives^24^, researchers from academia and industry call for efforts from multiple disciplines to develop models and algorithms to integrate the human reaction mechanisms into the development of effective safety systems^25^. Taken pedestrians for example, such research needs to precisely describe one of the most urgent tasks of the vehicle safety community, which is the construction of the safety boundaries of vehicle–pedestrian conflicts. In real-world accidental scenarios, about 66% pedestrians would perceive the traffic signals and foresee the emerging danger (e.g., collision by vehicle), make a decision, and react^26^. The kinematic features during reaction (e.g., moving velocity, acceleration) provide an important reference to ensure a possible optimal safe control on the vehicle side by providing collision avoidance or tailored protection devices (e.g., pop-up hood) in unavoidable collisions. In such context, virtual reality (VR)-based tools would allow naturalistic interactions of road users in risk-free environment and enable the controllability of the scenario conditions as evidenced in several preliminary attempts of use relevant to HAVs^27,28^.

To combine the emerging active safety requirements and the accumulated passive safety research, the present study aims to identify the safety envelope of pedestrians upon motor vehicle conflicts via pedestrian active behaviour feature. We proposed a novel VR-based experimental approach of pedestrians’ spontaneous kinematic signal measurement in an immerse, near-real traffic conflict scenario. The timeline of perception-decision-action, the underlying reaction mechanism and motion categories upon traffic emergency were presented. Safety envelopes of pedestrians in vehicle conflicts were estimated providing a reference for developing integrated safety systems to minimize human injury risk.

## Results

**Figure 1:**
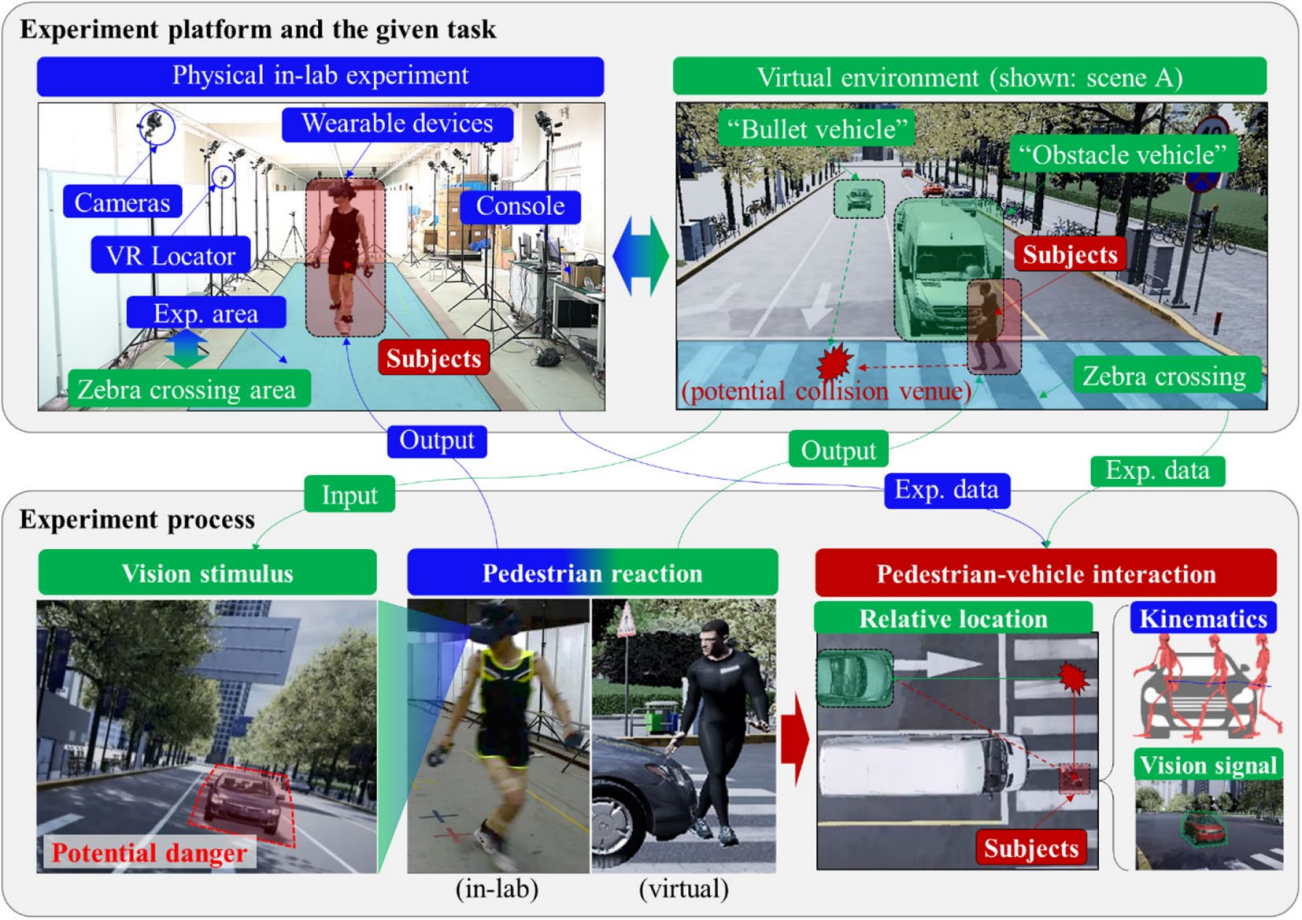
Overview of the experiment framework and the data flow (shown: lab experiment photos and captures of the virtual scene). In the virtual environment, “Obstacle vehicle” represents a vehicle that stopped in front of the zebra crossing and obstructed the pedestrian’s vision; “Bullet vehicle” represents a vehicle behind the obstacle vehicle that suddenly appeared and conflicted with the pedestrian.

We developed a mixed reality dynamic experimental environment with pre-defined, high-fidelity traffics scenes to generate audio-visual stimulus for subjects (“pedestrians”) in volunteer tests. The experiment environment is composed of a virtual test platform and a synchronously triggered signal capture module to generate near-real, immersive virtual traffic scenes with the parallel measurement of human kinematic signals (i.e., kinematics capture system) (Fig. 1, also see the Supplementary Movie S1 for detailed illustration). The in-lab experimental area covers about 100 m^2^, with the virtual experimental area (“Exp. area”) for pedestrians representing a 3.5-m-width and 13-m-length zebra crossing area. We performed a controlled experiment assigning subjects to complete the given traffic tasks, i.e., urban road crossing. Pedestrian reactions upon representative traffic conflicts with motor vehicles were examined based on the whole-body kinematics and relative motion to the vehicle in a real-time manner (see “Methods” section for data description).

### Categories of the natural pedestrian reactions

**Figure 2:**
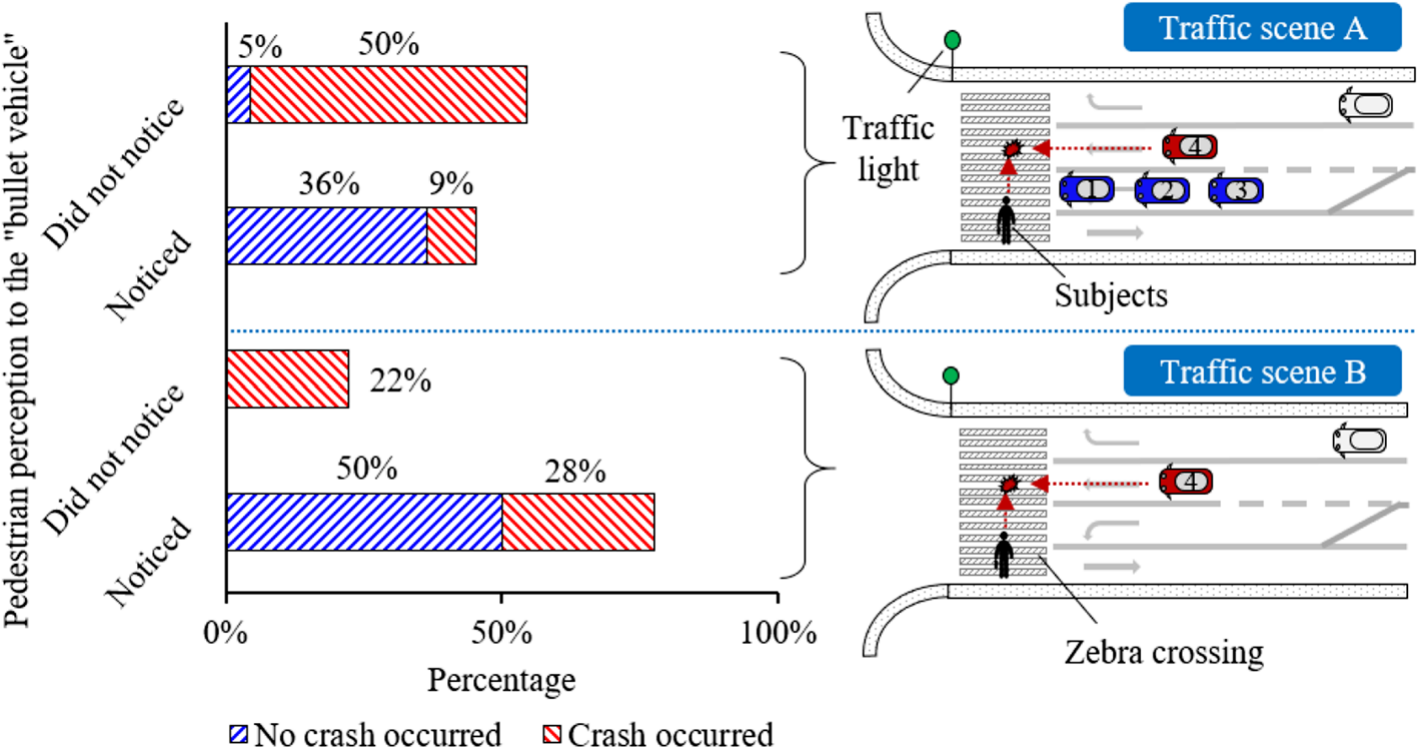
The distribution of the conflict results in two pre-defined traffic scene A and B (all figures produced by authors). Traffic scene A: road crossing with a hidden “bullet vehicle” (vehicle ④) upcoming behind visual obstacles (stopping vehicle ①, ②, ③); Traffic scene B: road crossing with the “bullet vehicle” (vehicle ④) upcoming without visual obstacles. “No crash occurred” denotes the cases with successful avoidance of the vehicle–pedestrian collision, “crash occurred” denotes the cases in which the collision between the virtual “bullet vehicle” and the pedestrians would not be not avoided.

Simultaneous reaction behaviour in 40 experimental cases performed by 22 subjects were recorded. Pedestrians exhibited a typical “perception-decision-execution” sequence along the timeline in two given typical traffic conflicts (i.e., crossing an urban road with and without visual obstacles, labelled as traffic scene A and B). Subjects mostly utilized vision for detecting the upcoming “bullet vehicle” and naturally took the avoidance behaviour with significantly activated muscles and moving kinematics. Such behaviour trend remains consistent in the two scenes but with different percentage of the population with efficient perception or execution (Fig. 2). In traffic scene A (22 cases), 12 subjects (55%) did not notice the “bullet vehicle” due to the “obstacle vehicle”, among whom 11 subjects (92% of the 12 cases) fell into vehicle–pedestrian collisions. In traffic scene B (18 cases), 14 subjects (78%) noticed the “bullet vehicle” and took avoidance reaction, among whom 5 subjects (36% of 14 cases) sustained “collision” due to avoidance failure.

Subjects exhibited normal walking, followed by four action categories in the vehicle–pedestrian conflicts. The visual, kinematics and location information of all subjects were completely synchronously recorded. The action categories were divided based on the pedestrian perception of the “accident vehicle” and the relative motion direction of the pedestrian body: (1) backward avoidance (BA) (13 cases, 33%) (i.e., pedestrians noticed the “bullet vehicle” and chose to move backwards for avoiding purpose; for the pedestrians who had not entered the vehicle lane, they would choose to stop); (2) forward avoidance (running) (FA) (9 cases, 23%) (i.e., rushing forwards); (3) oblique stepping (OS) (2 cases, 6%); (4) no avoidance reaction (NAR) (16 cases, 35%) (i.e., normal walking without noticing the upcoming vehicle; labelled as “collision occurred”). Pedestrians in the BA and FA categories noticed the coming vehicle, exhibited collision avoidance capability (85%, 67%) and sustained significant kinematic and posture change. The OS behaviour was essentially a startle response, where the pedestrians generally became overwhelmed and cannot avoid the collision.

### Vehicle–pedestrian interaction in the traffic conflicts

**Figure 3:**
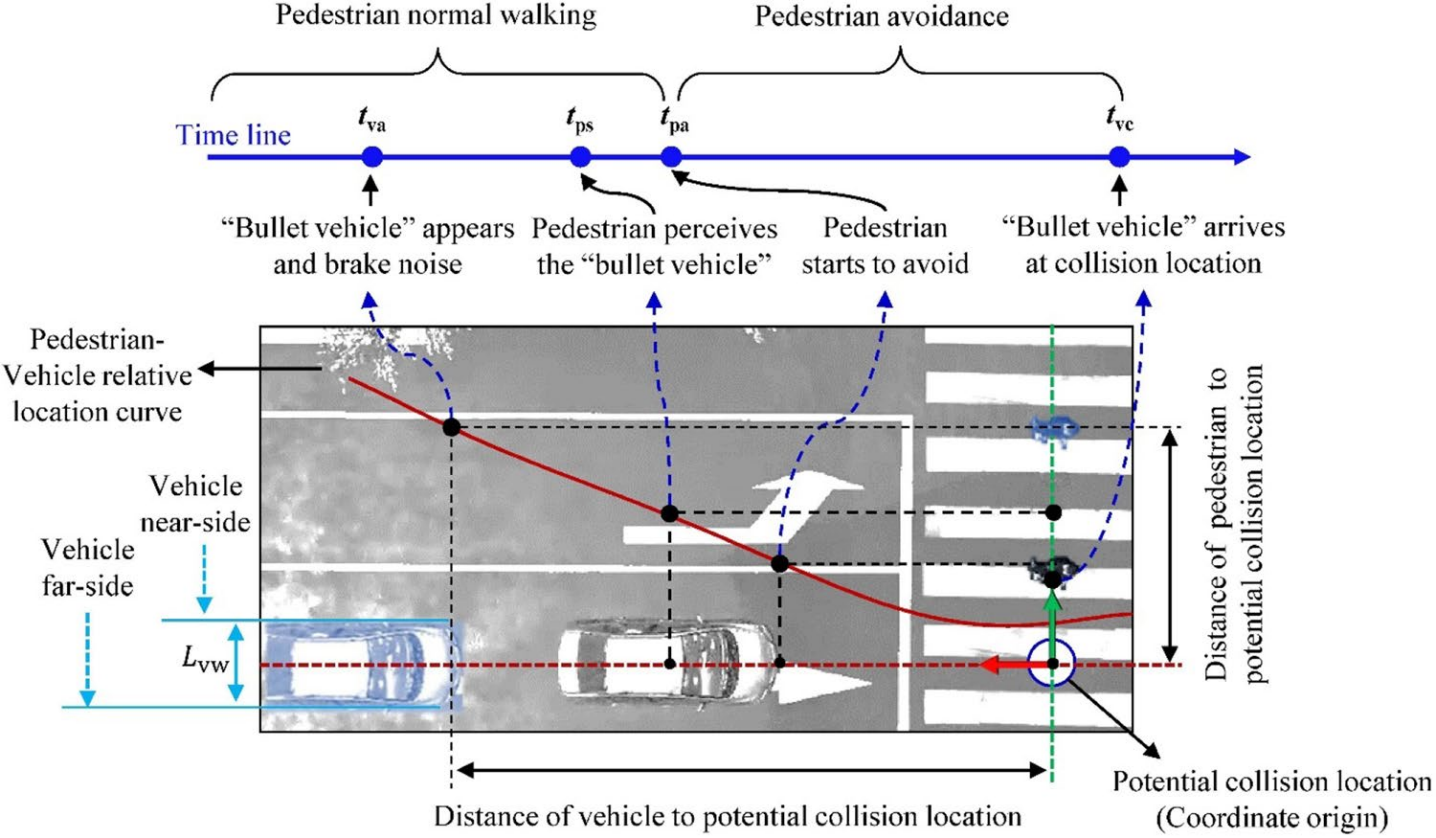
Vehicle–pedestrian interaction process identified in the experiments (shown: typical case in traffic scene B; produced by authors). Potential “collision venue” indicates the intersection of the initially given moving trajectories of the vehicle and the pedestrian. The horizontal and vertical axes indicate the distance of vehicle and pedestrian to the potential “collision venue”, respectively. The red curve was plotted with the potential “collision venue” as the origin to indicate the relative location of the pedestrian to the moving vehicle.

In both traffic scenes, the timing trends in the process of pedestrian avoidance remain consistent (Fig. 3). The whole event took approximately 2.2 s from the appearance of the danger to the vehicle arriving at the pre-designed “collision venue” (i.e., designated collision occurrence) (*t*_[*va*,_ _*vc*]_, traffic scene A: 2.22 ± 0.55 s, B: 2.20 ± 0.44 s). Pedestrians need more time to “perceive” the “bullet vehicle” (*t*_[*va*,_ _*ps*]_) in traffic scene A than in traffic scene B (average: 0.57 ± 0.29 s vs. 0.51 ± 0.27 s). The time gap between vision stimulation and moving reaction by pedestrian (*t*_[*ps*,_ _*pa*]_) in traffic scene A is less than that in the traffic scene B (average: 0.20 ± 0.18 s vs. 0.30 ± 0.28 s). Pedestrians need more time to perform “execution” process than “perception-decision” (*t*_[*pa*,_ _*vc*]_, traffic scene A: 1.45 ± 0.53 s, B: 1.37 ± 0.69 s).

**Figure 4:**
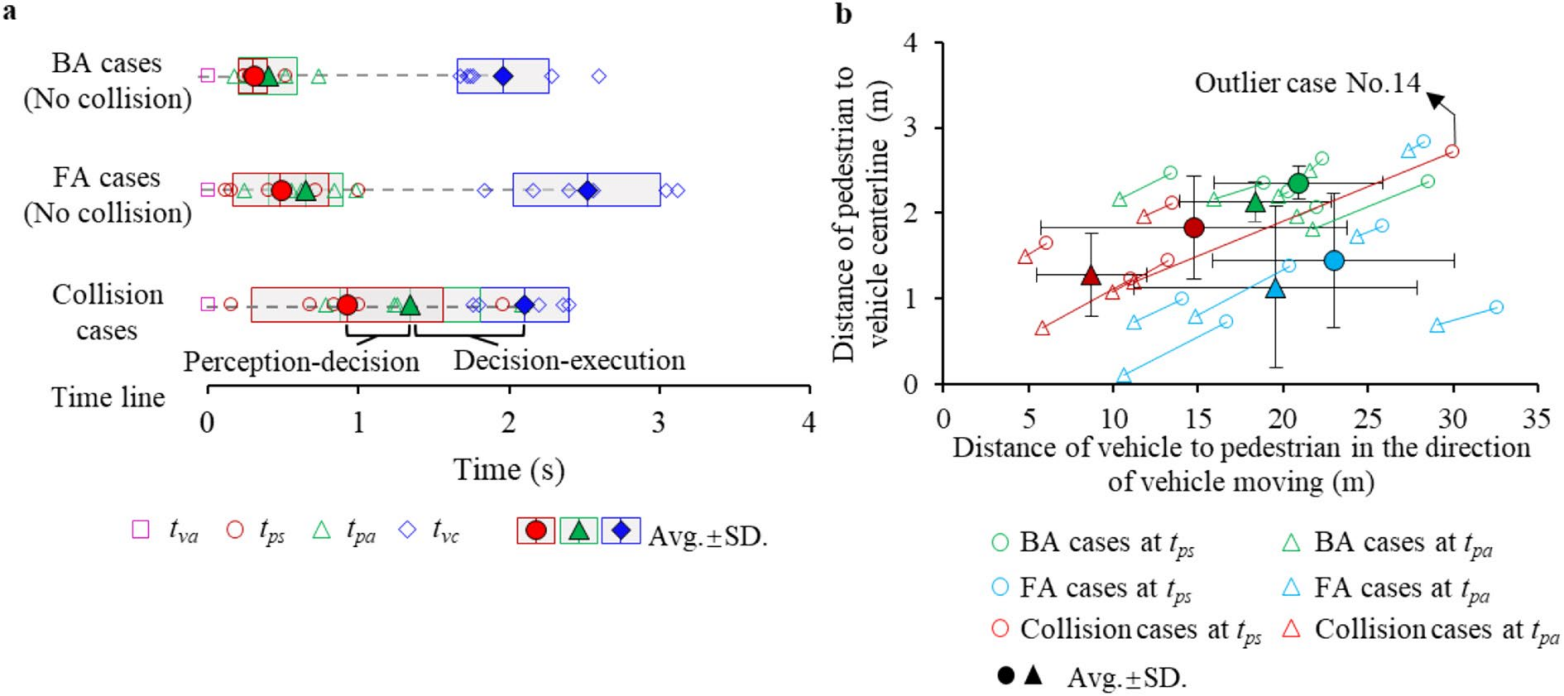
The avoidance process of pedestrians relative to the “bullet vehicle”: (**a**) the reaction timeline of pedestrian in the BA cases, FA cases (collision avoided) and collision cases subject to the manual trigger of the “bullet vehicle” by the experimenter; (**b**) the relative location of the pedestrian and the “bullet vehicle” at *t*_*ps*_ (danger perceived) and *t*_*pa*_ (decision made); the same case is connected by a solid line; the centre of the vehicle front-end is taken as the coordinate origin.

For the successful avoidance cases in BA and FA categories, the BA behaviour took overall less “perception-decision-execution” time than the FA behaviour (Fig. 4a), with both shorter “decision” time (*t*_[*ps*,_ _*pa*]_, 0.17 ± 0.14 s vs. 0.24 ± 0.17 s) and “execution” time (*t*_[*pa*,_ _*vc*]_, 1.52 ± 0.36 s vs. 1.84 ± 0.64 s). The distances between the pedestrian and the “collision venue” at *t*_*pa*_ and *t*_*ps*_ in the BA behaviour are longer than the FA behaviour (Fig. 4b). That is, upon perceiving the potential danger from the “bullet vehicle”, the pedestrians who initially located closer to the vehicle lane are more likely to choose FA behaviour. For the collision cases, the “decision” time of pedestrians is longer than that in the successful avoidance cases (i.e., *t*_[*ps*,_ _*pa*]_, collision cases: 0.41 ± 0.40 s, BA: 0.17 ± 0.14 s, FA: 0.24 ± 0.17 s). The long “decision” time led the pedestrians located close to the “bullet vehicle” and not having enough time to execute avoidance behaviour (i.e., *t*_[*pa*,_ _*vc*]_, collision cases: 0.76 ± 0.31 s, BA: 1.52 ± 0.36 s, FA: 1.84 ± 0.64 s). In the one outlier (case No. 14, Supplementary Table 1) (Fig. 4b), the subject made an early perception but a relatively late decision on avoidance motion of forwards move, and finally sustained a virtual collision.

### Characterizing pedestrian avoidance ability by whole-body kinematics

**Figure 5:**
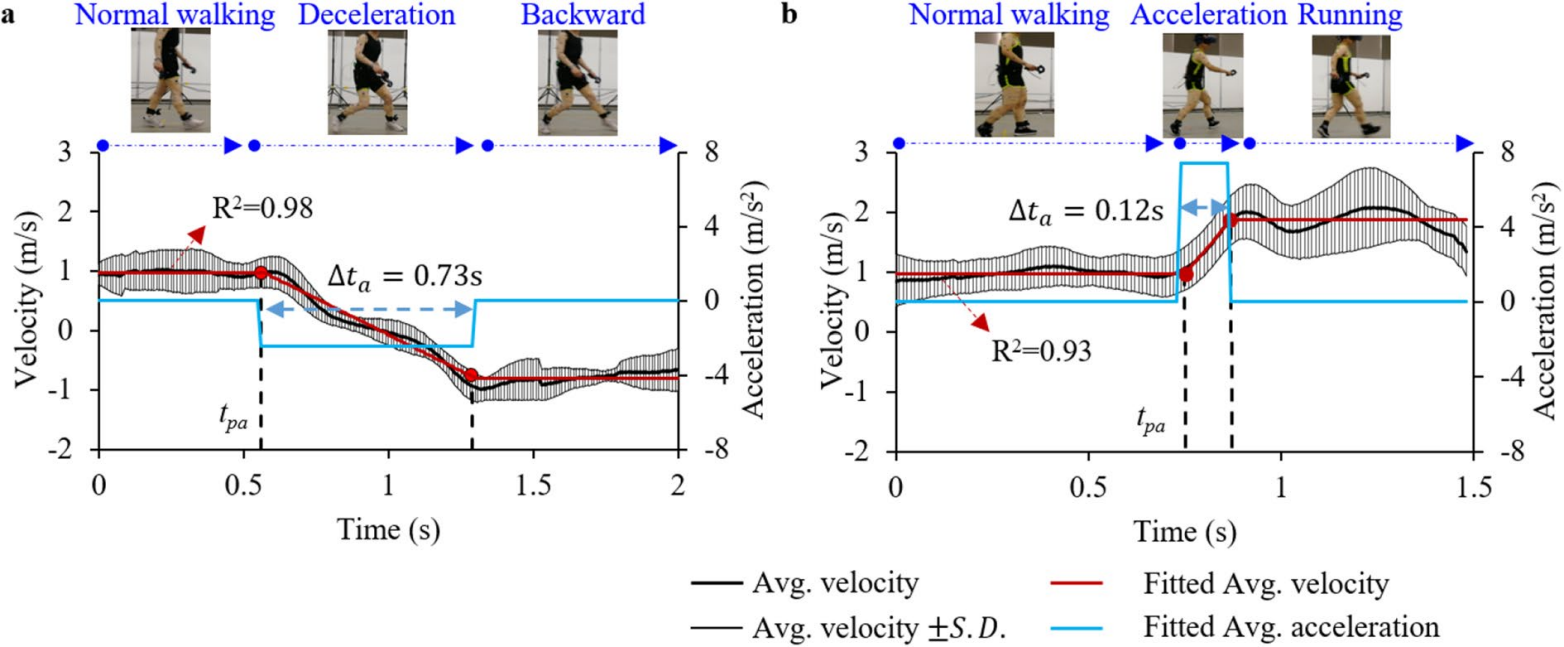
The whole-body kinematics of the pedestrian in the avoiding process of “bullet vehicle” by (**a**) backward and (**b**) forward motion. $\Delta {t}_{a}$ denotes the duration of acceleration or deceleration (shown: lab experiment photos).

Subjects would activate the natural avoidance ability once they perceive the upcoming vehicles. The whole-body kinematics in the process of avoiding danger indicate the trend of the natural avoidance ability by the pedestrian. The BA behaviour by a pedestrian who entered the vehicle lane generally exhibited a three-phase motion: normal walking with an initial velocity of approximately 1 m/s, uniformly decelerating over approximately 0.73 s (on average, *a*_p.BA_  = − 2.4 m/s^2^), followed by a backward move with an − 1 m/s velocity (Fig. 5a). Differently, pedestrians in the FA category exhibited a complete forward accelerating (on average, *a*_p.FA_ = 7.4 m/s^2^) to approximatively 1.8 m/s and running forward for avoidance purpose, with an acceleration process of 0.12 s on average (Fig. 5b). In each case, the activation time (*t*_*pa*_) was identified from the measured velocity curves and was double-checked by manually reviewing the recorded video information to ensure correctness.

### Estimated average safety envelope based on the collision avoidance capability of the pedestrian and the vehicle

**Figure 6:**
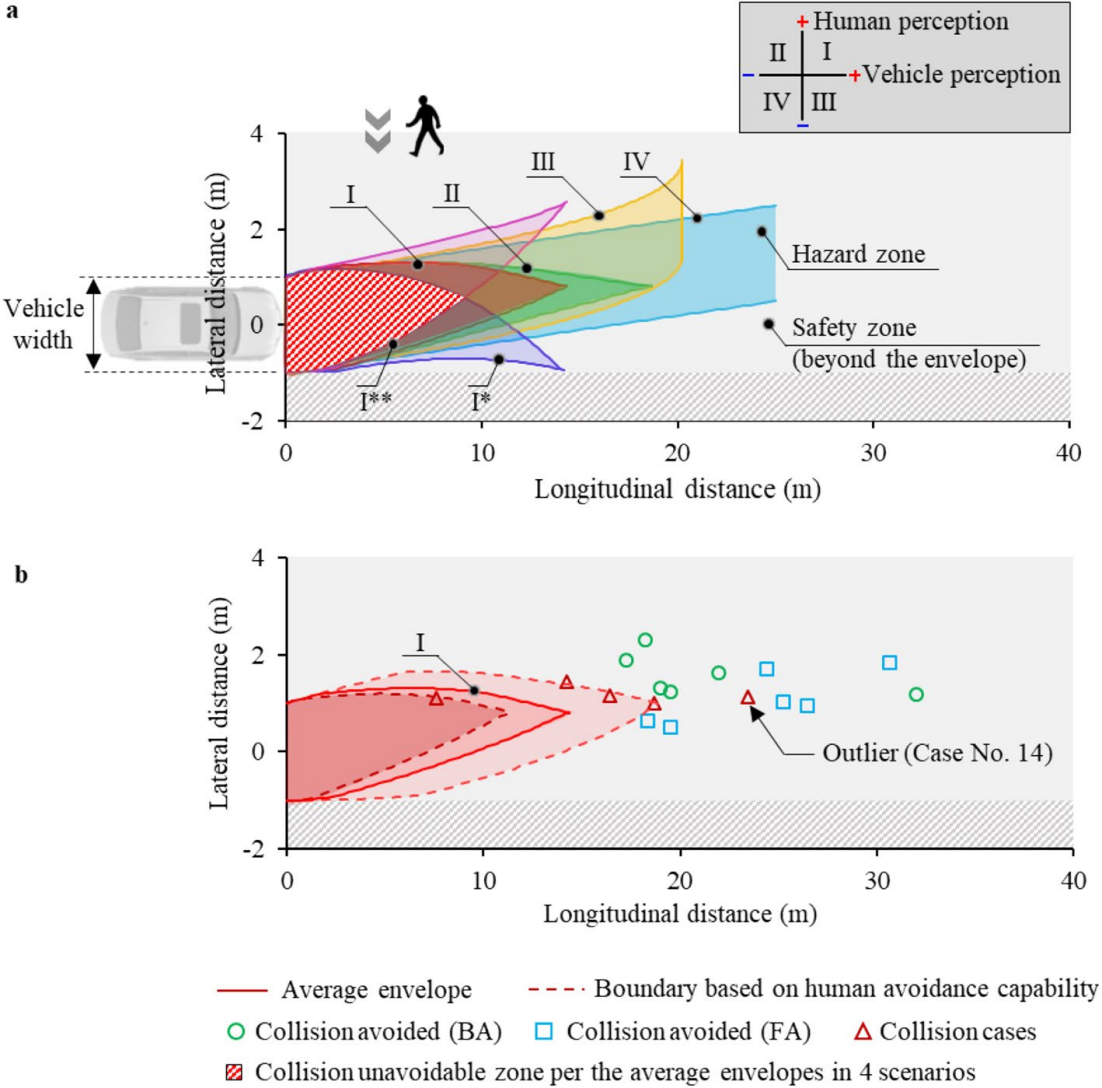
(**a**) Safety envelope of pedestrians upon vehicle collisions under different vehicle–pedestrian interaction conditions; the shaded hazard zones indicate the initial pedestrian locations where collisions are unavoidable with a normal driving vehicle; (**b**) safety boundary based on human avoidance capability.

We proposed four typical vehicle–pedestrian interaction scenarios (I, II, III, IV) distinguished by awareness capability of the pedestrian and the vehicle (including driver and/or detection device) to each other (see the “Methods” and the Supplementary Section II for detailed derivation and results). In each condition, an analytical safety envelope was estimated to identify relative safe and hazard zones between vehicle and pedestrian using estimated pedestrian kinematics and vehicle motion (Fig. 6a). Pedestrians who are initially located in the labelled hazard zone (shaded) would sustain unavoidable collisions.

Under scenario (I), both the pedestrian and vehicle notice each other. Pedestrians would be moving to the “collision venue” with natural avoidance behaviour; the vehicle would be moving to the “collision venue” at maximum braking. If steering to the left/right is further included as an additional collision avoidance measure on the vehicle side, the maximum possible steering trajectory can be estimated (Fig. 6a, I*, I**). The overlapped area of the plotted scenarios indicates “collision unavoidable” zones under the present traffic conflicts.

Scenario (I) agrees with the present experimental setup, and distribution of the experimental results is provided in the same scale (Fig. 6b). The upper and lower boundary of the envelope was determined to illustrate the maximum (minimum) avoidance ability using scenario (I) as one example (the range of the reaction time and avoidance velocity are in reference to the experimental results presented in Figs. 4 and 5). Most experiment cases agreed with the safety envelope derived from the analytical estimation. In the only outlier (the same outlier case as in Fig. 4b), the subject made a “mistake” decision and sustained a virtual collision.

## Discussion

Identifying natural human reaction to emergency and potential harms is essential to safety decisions made under uncertainty. An interesting question is, to what utmost extent the safety systems can be developed utilizing the naturalistic danger-avoidance behaviour of human. As a first investigation, we used immersive virtual reality technology for providing near-real traffic scenarios and simultaneous motion caption system for recording the human reaction and applied the experimental data to develop the safety envelopes. Recognition of the physiological effects through the representative scenarios also renders future safety applications practical.

The present study observed that the natural human response in potential traffic danger can be divided into several categories according to kinematic information (Fig. 4). For example, the subjects in traffic scene A cannot observe all road environment due to the in-purposely embedded “obstacle vehicle” (Fig. 2), resulting in a high proportion of collision cases (i.e., 59%). Differently in scene B, the subjects who could observe all surroundings remained high safety-conscious and executed avoidance behaviour, resulting in a 50% self-avoidance of collision and a 28% of collision failure, where the latter were mostly due to limited reaction time. The generated results are expected to provide evidence on biomechanical features for traffic safety investigation during or before motor vehicle collisions, such as referenced pre-collision posture for physical safety testing and computational investigation using mathematical pedestrian models^29^. With the currently wide use of modelling, development of biofidelic human models (such as these with active responses) would further contribute to the generation of a large enough databank of human response surface in traffic safety research.

For engineering applications, the finding on human reactions is an important reference in the development of advanced vehicle safety systems. A majority of existing on-board active safety systems employ emergency braking measures for vehicles (e.g., Autonomous Emergency Braking, AEB^30,31^). Yet, braking only is not always the best action for vehicle given the active movement of the pedestrian itself. Proper steering with motion prediction of the pedestrian reduces the hazard zones (Fig. 6) and is potential to avoid real collisions especially for highly automated transportation tools capable of sensing pedestrian information ahead of time^32^. Towards such applications, our results indicated a time gap of about 0.17–0.41 s (i.e., *t*_[*ps*,_ _*pa*]_) from human perceiving signal to execution (Fig. 4a), which is comparable to the previous studies^13^. The activation of the pedestrian “execution” can be identified and predicted via kinematic feature (e.g., velocity change). As velocity is one of the most significant influencing factors on injury probability and severity in motor vehicle collisions^7^, the subsequent collision consequences can be more precisely predicted and better handled by incorporating realistic pedestrian response.

We demonstrated the use of pedestrian reaction mechanisms in safety envelopes upon conflicts by combining the collision avoidance capability of the human and the parallel motion of the vehicle. By using a straightforward approach, the average envelope under typical scenarios was plotted as an example via analytical estimation. The boundaries of such envelopes would be influenced by both pedestrian behaviour (e.g., moving kinematics) and vehicle dynamics (e.g., braking capability) as evidenced by the experimental data (Fig. 6). Understanding the most representative reaction of pedestrians can contribute to the development of protective system concerning pedestrian safety. The proposed safety envelopes provide two guidelines: (1) implementing the safety envelope into on-board system on vehicles to detect the pedestrian state, predict the pedestrian motion and identify whether pedestrians are in the hazard zones, and (2) developing tailored passive protection of pedestrian in unavoidable collisions to reduce the injury risk in collisions. The former shall be achieved by active safety system: once the sensor information is sufficient to detect the motion information of the pedestrian on road, the developed safety envelope can provide a rapid prediction to avoid the potential collisions. This prediction process reduces the computational burden and is essential for real-world applications. The latter was shifted to an optimal design against unavoidable collisions via protection structure, which can be realized in the use of accumulated passive safety research in past decades. Possible measures include absorbing kinetic energy in mitigating collision severity (e.g., vehicle hood with sandwich inner^33^ or with pedestrian airbag^34^).

Yet, it shall be noticed that the present study has several limitations. First, the investigation was limited to the given traffic scenario, which although deems one most representative vehicle–pedestrian conflict. To how much wider coverage the observed reaction trends remain valid is not quantified and shall be verified with diverse traffic scenarios. Second, we restricted the test subjects to young, average adults to avoid populational factors in this pilot investigation. Other vulnerable pedestrian populations, such as the elderly and the children, sustain high injury risk in vehicle collisions^35,36^ and need to be further considered. More detailed physiological data (e.g., visual direction) shall be collected and used to study the pedestrian emergency reaction time and human active response, and the study is expected to be continuously improved based these aspects.

To the best of our knowledge, this study represents the first attempt of fusing natural pedestrian behaviour facing the development needs of effective safety systems (e.g., for vehicles of high automation). It demonstrates the feasibility of using real-time unobtrusive measurement of pedestrian and the behavioural effects for providing a straightforward safety prediction. By exploring the natural response of human, and by combining human responses with robotic systems and their interactions, it opens the way for future engineering design and collision mitigation algorithm which can facilitate improved, interactive devices for a global optimal safety on road.

## Methods

### A mixed reality dynamic experimental environment

#### Virtual test platform

Generation of virtual scene was performed on a high-fidelity VR simulator manufactured by 51VR High Technology Co., LTD. A well-defined traffic environment based on a four-lane intersection was provided for the experiment subjects to enter (resolution of the virtual scene rendering: 2800 × 1600, rendering rate: 91 FPS, rendering delay: < 11 ms). The built-in traffic elements in the virtual environment create a strong sense of reality, including infrastructure, buildings, trees, traffic light, vehicle, surrounding pedestrians, traffic noise etc. Subjects entered the virtual environment as “pedestrians” with wearable VR devices for signal recording (HTC Vive Pro with wireless adapter). A control system module was designed to control the interaction between the human-in-loop and the virtually implemented traffic objects, including the appearance of the “bullet vehicle”, neighbouring pedestrians and traffic signal conversion. Posture and spatial position of the subject body were tracked, monitored, and translated into the VR space in real-time by VR locators. The location of the subject during the human-in-loop interaction was also recorded (sampling frequency: 25 Hz).

#### Kinematics capture system

The kinematic capture system (No. Mars 2H; Beijing Nokov Science & Technology Co., Ltd) recorded the key motion information of subjects during the experiments. Setup of the kinetic capture system consists of 12 cameras, 54 on-body markers and a software module. The cameras (sampling frequency: 100 Hz) were fixed on the edge of the subjects’ movement range by tripods. Motion of the subject throughout the whole event was recorded using 54 markers adhered externally to the skin on body. Algorithm processing of the kinematic images yielded signals of the quantified whole-body kinematics. Feature velocity of the pedestrian was extracted at the centre of the pelvis.

### Volunteer test

#### Subjects and ethics statement

Participating subjects were recruited on campus, Qinghuayuan communities (population about 100,000) through flyer posting physically and online via WeChat Group (i.e., the largest online social platform in China). Subjects had normal (or corrected to normal) vision, hearing, and walking gait, without disability or heart disease. For sampling, subjects were selected as a representative of normal adult, young cohort to avoid populational factors. We restricted admission to male individuals who were between 18 and 30 years of age. Data for *n* = 22 subjects (age range 22.0 ± 1.8 years; height range 174.0 ± 4.0 cm; BMI range 21.9 ± 2.4) were complete and finally included in the data analysis. Informed consent from each test subject was obtained before conducting the experiments. For recording a real reaction upon traffic conflicts, subjects were told that the study purpose was to investigate the street crossing behaviour while remained unaware of the simulated vehicle–pedestrian conflict. The subjects could stop the experiment at any time if they had any discomfort during the experiment. The experimental procedures were approved by the Institutional Review Boards (IRB) of Tsinghua University. All experiments were performed in accordance with relevant guidelines; the informed consent to publish identifying information and images obtained from all individual participants were included in the study.

#### Test procedure and data

Two pre-defined traffic scenes were provided in sequence to each subject to produce a vehicle–pedestrian interaction using the programmed behaviour of a specific vehicle (Fig. 2). At the beginning of the experiment, a trigger signal would start each device synchronously to record information. We programmed the VR devices to save a record of the vehicle parameters, including speed, acceleration, relative position of the subject (“pedestrian”) to the vehicle. For each case, the experimental process is divided into 3 phases:

Phase 1 (Warm-up): The subjects familiarized themselves with the traffic scenarios on side of a four-lane urban street for 2 min. Following this, the subject was told to wait and to cross the road when traffic signals on the other side turned green. Regular vehicle flow was provided for a near-real scene before the signals turned green.

Phase 2 (Normal traffic): Prior to the signals turning green, three vehicles (only in traffic scene A) would stop in the second lane in front of the subject, and in-purpose obstructed the subject’s view to the third lane. Subjects confirmed safety scene and began to cross the zebra crossing with a normal walking velocity.

Phase 3 (Occurrence of traffic conflict): Right after the subject stepped in the middle of the second lane, a vehicle–pedestrian conflict was made by the experimenter: a pre-defined accident vehicle (“bullet vehicle”) on the third lane had some “malfunction”, propelling itself forward and resulting into an unintended rushing laterally to the subject. The “bullet vehicle” was set to run the red light by an initial velocity of 80 km/h and a braking deceleration of 0.7 g. The arrival timings of the “bullet vehicle” to the potential collision venue of all cases (*t*_*vc*_) lie within a 0.5 s time window due to the manual operation of the experimenter (Fig. 4). Subjects who noticed the “bullet vehicle” would naturalistically exhibit self-avoidance behaviour; otherwise, the subjects who paid no attention to the “bullet vehicle” and kept normal walking would move to the potential “collision venue” and encounter a virtual collision. Reaction and kinematics of subjects during the conflicts were extracted as feature data using VR control software and kinematic capture system in the experimental phases.

### The safety envelope developed with the pedestrian avoidance ability

**Figure 7:**
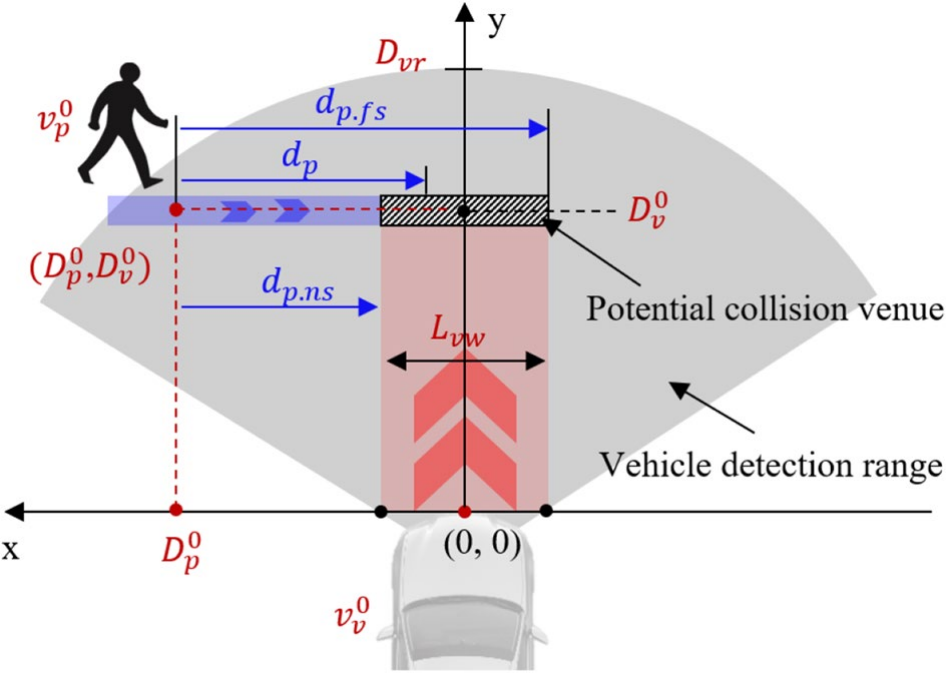
Illustration of the pedestrian motion in the on-vehicle coordinate system. The centre of the vehicle front-end is taken as the origin. ${D}_{\text{vr}}$ denotes the radius of the detection range for typical vehicle sensing systems.

We estimated the critical zones indicating collision possibility (i.e., safety envelope) using the instant relative vehicle–pedestrian distance in both the longitudinal (*y*) and lateral directions (*x*) within on-vehicle coordinate system (Fig. 7). An analytical envelope was calculated based on the motion of the pedestrian and the vehicle under the experimental traffic scenario. A pedestrian initially located within the distance-based envelope (labelled as “hazard zone”) for a vehicle would sustain unavoidable collisions. At a given moment of time *t*, for the pedestrians who did not notice the “bullet vehicle”, they would maintain normal walking during the road-crossing traffic task.

1 $${v}_{p_{\cdot} a}\left(t\right)={v}_{p}^{0}$$

where ${v}_{p_ {\cdot} a}$ is the pedestrian avoidance velocity during interaction between of pedestrian and vehicle, ${v}_{p}^{0}$ is the pedestrian initial normal walking velocity.

For the pedestrians with activated avoidance ability, the avoidance behaviour (i.e., from visually notice the upcoming vehicle) can be divided into two phases: “perception-decision” (*t*_[*ps*,_ _*pa*]_) and “decision-execution” (*t*_[*pa*,_ _*vc*]_) (Fig. 4), the “decision-execution” phase can be further divided into acceleration phase and uniform motion phase (Fig. 5). Therefore, the avoidance velocity can be simplified along the straight line of the pedestrian initial moving direction.

2 $${v}_{p_ {\cdot} a} \left(t\right)=\left\{\begin{array}{l}{v}_{p}^{0}, t\in \left(0, {\Delta t}_{r}\right)\\ {v}_{p}^{0}+{a}_{p_ {\cdot} a}{(t-\Delta t}_{r}), t\in \left({\Delta t}_{r}, {\Delta t}_{r}+{\Delta t}_{a}\right)\\ {v}_{p}^{0}+{a}_{p_ {\cdot} a}\Delta {t}_{a}, t\in \left({\Delta t}_{r}+{\Delta t}_{a},+ \infty \right)\end{array}\right.$$

where ${\Delta t}_{r}$ is the pedestrian reaction time (i.e., from perceiving the “bullet vehicle” to start avoidance, *t*_[*ps*,_ _*pa*]_); ${a}_{p_ {\cdot} a}$ is pedestrian acceleration during the avoidance phase; ${\Delta t}_{a}$ is the duration of acceleration or deceleration in the process of avoidance (Fig. 5).

To be realistic, only crossing pedestrians within the detection range of sensor system on vehicle are considered. At the beginning of the traffic event, the lateral distance between the pedestrian and the vehicle is estimated as

3 $${d}_{p_ {\cdot}ns}=-\frac{{L}_{vw}}{2}+{D}_{p}^{0}$$

4 $${d}_{p_ {\cdot} fs}=\frac{{L}_{vw}}{2}+{D}_{p}^{0}$$

where ${d}_{p_ {\cdot}ns}$ and ${d}_{p_ {\cdot} fs}$ denote the distance between the pedestrian and the near or far side of the vehicle, ${D}_{p}^{0}$ is the lateral distance of pedestrian to vehicle centreline, ${L}_{\text{vw}}$ is the vehicle width.

At the time when the vehicle arrives the designated “collision venue”, if the moving distance of the pedestrian, ${d}_{p}$, is within [${d}_{p_ {\cdot} ns}$, ${d}_{p_ {\cdot} fs}$], the subsequent collision is denoted as unavoidable, and vice versa:

5 $${d}_{p}({t}_{v})= {\int }_{0}^{{t}_{v}}{v}_{p_ {\cdot} a}(t)dt$$

6 $$\text{Collision occurs},\text{ if }{d}_{p_ {\cdot}ns}\le {d}_{p} \le {d}_{p_ {\cdot}fs}$$

where ${t}_{v}$ is the time of vehicle arriving at the potential “collision venue”, ${d}_{p}$ is the pedestrian moving distance during avoiding within ${t}_{v}$.

Thus, the occurrence criterion for vehicle–pedestrian collision can be converted as a ${d}_{p}$-based safety envelope.

7 $$-\frac{{L}_{vw}}{2}+{d}_{p}({t}_{v})\le {D}_{p}^{0}\le \frac{{L}_{vw}}{2}+{d}_{p}({t}_{v})$$

## Supplementary Information

Supplementary Information 1.

Supplementary Video S1.

## Data Availability

The datasets generated and analysed during the current study are available from the corresponding author on reasonable request.
