# Supplementary material for: Safety envelope of pedestrians upon motor vehicle conflicts identified via active avoidance behaviour
Source: Sci Rep. 2021 Feb 17;11:3996. doi: 10.1038/s41598-021-82331-z (PMC7889901; doi:10.1038/s41598-021-82331-z)
Supplement: Supplementary file 1 — Supplementary Information 1. [file 41598_2021_82331_MOESM1_ESM.pdf]

Supplementary Materials

Safety envelope of pedestrians upon motor vehicle conflicts identified via active avoidance behaviour

Bingbing Nie[1]*, Quan Li[1], Shun Gan[1], Bobin Xing[1], Yuan Huang[1], Shengbo Eben Li[1]

[1]State Key Laboratory of Automotive Safety and Energy, School of Vehicle and Mobility, Tsinghua University, Beijing 100084, China

* nbb@tsinghua.edu.cn

I. Experimental data

Supplementary Table 1. Reaction time and relative distance from the vehicle in the cases where the pedestrians perceived the coming bullet vehicle before collision. Visual, kinematics and location information of 17 complete experimental cases were presented. $D_p(t_{ps})$, $D_p(t_{pa})$, $D_v(t_{ps})$, $D_v(t_{pa})$ and $v_p(t_{ps})$, $v_p(t_{pa})$, $v_v(t_{ps})$, $v_v(t_{pa})$ represent the the relative location and moving velocity of the pedestrian and the "bullet vehicle" at t_{ps} (danger perceived) and t_{pa} (decision made), with the centre of the vehicle front-end taken as the origin.

BA cases (No collision cases included)											
Case No.	t_{ps} (s)	t_{pa} (s)	t_{vc} (s)	$D_p(t_{ps})$ (m)	$D_v(t_{ps})$ (m)	$D_p(t_{pa})$ (m)	$D_v(t_{pa})$ (m)	$v_p(t_{ps})$ (m/s)	$v_v(t_{ps})$ (km/h)	$v_p(t_{pa})$ (m/s)	$v_v(t_{pa})$ (km/h)
01	0.32	0.56	1.74	2.34	18.87	2.17	15.91	1.01	61.7	0.92	58.5
02	0.28	0.32	2.6	2.64	22.33	2.5	21.57	2.14	41.8	2.07	41.4
03	0.24	0.28	1.68	2.25	20.35	2.2	19.75	1.56	64.3	1.52	63.7
04	0.18	0.24	1.72	2.07	22.02	1.96	20.8	1.61	67.6	1.51	66.1
05	0.28	0.52	1.76	2.47	13.41	2.17	10.34	1.28	46.6	1.12	41.9
06	0.32	0.74	2.28	2.36	28.56	1.82	21.72	1.41	78.1	1.23	68.9
Avg.	0.27	0.44	1.96	2.36	20.92	2.14	18.35	1.50	60.0	1.40	56.7
SD.	0.05	0.18	0.35	0.18	4.52	0.21	4.08	0.38	13.5	0.40	12.2

FA cases (No collision cases included)											
Case No.	t_{ps} (s)	t_{pa} (s)	t_{vc} (s)	$D_p(t_{ps})$ (m)	$D_v(t_{ps})$ (m)	$D_p(t_{pa})$ (m)	$D_v(t_{pa})$ (m)	$v_p(t_{ps})$ (m/s)	$v_v(t_{ps})$ (km/h)	$v_p(t_{pa})$ (m/s)	$v_v(t_{pa})$ (km/h)
07	0.66	0.72	3.04	2.84	28.3	2.73	27.4	1.51	55.5	1.55	54.7
08	0.52	0.98	1.84	0.72	16.7	0.1	10.62	1.33	51.4	1.48	46.9
09	0.4	0.62	3.12	0.9	32.6	0.69	29.1	0.91	74.1	0.97	69.3
10	0.12	0.56	2.16	1.38	20.39	0.8	14.87	1.25	48.4	1.41	43.2
11	0.84	1	2.4	0.99	14.07	0.73	11.2	1.46	45.9	1.55	11.9
12	0.16	0.24	2.56	1.84	25.85	1.73	24.32	1.05	63.5	1.11	61.5
Avg.	0.45	0.69	2.52	1.45	22.99	1.13	19.59	1.25	56.4	1.35	47.9
SD.	0.28	0.28	0.50	0.79	7.14	0.94	8.33	0.23	10.6	0.24	20.0

Collision cases											
Case No.	t_{ps} (s)	t_{pa} (s)	t_{vc} (s)	$D_p(t_{ps})$ (m)	$D_v(t_{ps})$ (m)	$D_p(t_{pa})$ (m)	$D_v(t_{pa})$ (m)	$v_p(t_{ps})$ (m/s)	$v_v(t_{ps})$ (km/h)	$v_p(t_{pa})$ (m/s)	$v_v(t_{pa})$ (km/h)
13 (FA)	0.84	1.34	2.2	1.22	11.08	0.66	5.82	1.00	40.1	1.19	31.4
14 (BA)	0.16	1.24	1.8	2.72	30	1.2	11.25	2.3	77.1	1.18	57.9
15 (BA)	1.00	1.26	2.36	1.44	13.2	1.08	9.99	1.38	45.4	1.25	40.8
16 (FA)	1.96	2.08	2.4	1.65	6.08	1.5	4.78	1.53	46.8	1.68	45.2
17 (FA)	0.68	0.78	1.76	2.12	13.47	1.96	11.78	1.42	56.3	1.48	53.9
Avg.	0.93	1.34	2.10	1.83	14.77	1.28	8.72	1.52	53.1	1.35	45.8
SD.	0.66	0.47	0.31	0.60	9.02	0.49	3.21	0.47	14.6	0.21	10.5

II. Estimation of the distance-based safety envelopes

We propose four simplified conditions (I, II, III, IV) of pedestrian-vehicle interaction depending on the awareness of the pedestrian and the vehicle (driver or sensor) of each other. In each condition, the hazardous zones can be determined

by path prediction of the vehicle and the pedestrian. According to the experimental results, the pedestrian avoidance ability was defined based on the average acceleration, duration time of acceleration and reaction time in the avoidance process (Figure 4, 5; Supplementary Table 2). We made an example to show the calculation result of safety envelope; the distance-based safety envelope was calculated based on the average pedestrian avoidance ability and a group of representative parameters of the vehicle and the pedestrian (Figure 6; Supplementary Table 3).

Supplementary Table 2. Average pedestrian avoidance ability

Pedestrian behaviour	$a_{p.a}$ (m/s^2)	Δt_a(s)	Δt_r(s)
Backward avoidance	-2.4	0.73	0.17
Forward avoidance	7.4	0.12	0.24

where $a_{p.a}$ is the pedestrian avoidance acceleration (Figure 4 in the main text).

Supplementary Table 3. Assumptions of the representative interaction conditions between the vehicle and the pedestrian detected by vehicle sensing system.

Vehicle parameters					
L_{vw}(m)	$a_{v.a}$(g)	$a_{v.al}$(g)	v_v^0(km/h)	D_{vr}(m)	D_v^0(m)
2	-0.7	-0.65	60	100	(0, D_{vr})
Pedestrian parameters					
v_p(m/s)	Moving direction				
1.0	From the front-left of vehicle and vertical to vehicle moving direction				

where $a_{v.a}$ is the maximum longitudinal acceleration of the vehicle; $a_{v.al}$ is the maximum lateral acceleration; v_v^0 is initial moving velocity, D_v^0 is the distance between the vehicle and the potential collision venue (Figure 7 in the main text).

The four interaction conditions and the calculation of the safety envelope are explained as below:

(I) Both the pedestrian and vehicle notice each other: pedestrians would be moving to the "collision venue" with natural avoidance behaviour; vehicle would be moving to the "collision venue" at the maximum braking (no steering included).

Solving the time of vehicle to potential collision venue (t_v) based on the initial velocity (v_v^0), braking deceleration ($a_{v.a}$) and the distance from vehicle to potential collision venue (D_v^0) (Figure 7).

$$D_v^0 = v_v^0 \cdot t_v + 0.5 \cdot a_{v.a} \cdot t_v^2 \quad \text{(S1)}$$

For the backward avoidance, backward avoidance velocity ($v_{p.aBA}$) and distance ($d_{p.aBA}$) of the pedestrian during avoidance can be determined based on its moving velocity (eq. 2) and the pedestrian-vehicle interaction (Supplementary Table 2).

$$v_{p.aBA}(t) = \begin{cases} v_p^0, \ t \in (0, \Delta t_r) \\ v_p^0 + a_{p.a}(t - \Delta t_r), \ t \in (\Delta t_r, \ \Delta t_r + \Delta t_a) \\ v_p^0 + a_{p.a}\Delta t_a, \ t \in (\Delta t_r + \Delta t_a, +\infty) \end{cases} \quad \text{(S2)}$$

$$d_{p.aBA}(t_v) = \int_0^{t_v} v_{p.aBA}(t)dt \quad \text{(S3)}$$

where $v_{p.aBA}$ represents the velocity of the pedestrian during backward avoidance, $d_{p.aBA}$ represents the pedestrian moving distance in the process of backward avoidance.

For the forward avoidance, forward avoidance velocity ($v_{p.aFA}$) and avoidance distance ($d_{p.aFA}$) of the pedestrian during avoidance can be determined based on its moving velocity (equation (2) in the main text) and the pedestrian-vehicle interaction (Supplementary Table 2).

$$v_{p.aFA}(t)=\begin{cases} v_p^0,\ t \in (0, \Delta t_r) \\ v_p^0 + a_{p.a}(t - \Delta t_r),\ t \in (\Delta t_r,\ \Delta t_r + \Delta t_a) \\ v_p^0 + a_{p.a}\Delta t_a,\ t \in (\Delta t_r + \Delta t_a, +\infty) \end{cases} \tag{S4}$$

$$d_{p.aFA}(t_v) = \int_0^{t_v} v_{p.aFA}(t)dt \tag{S5}$$

where $v_{p.aFA}$ represents the velocity of pedestrian during forward avoidance, $d_{p.aFA}$ represents the distance of pedestrian moving in the process of forward avoidance.

Determining the hazard distance of the pedestrian to the centreline of the vehicle (equation (S1)-(S5) and (5)-(7) in the main text):

$$-\frac{L_{vw}}{2} + d_{p.aBA} \le D_p^0 \le \frac{L_{vw}}{2} + d_{p.aFA} \tag{S6}$$

Under scenario (I), vehicle steering can be further included as an additional collision avoidance measure on the vehicle side. Following this, the maximum possible steering trajectory of the vehicle can be estimated. We use scenario (I*) to denote the condition that vehicle would be moving to the "collision venue" at maximum braking and maximum right steering. Vehicle velocity (v_v) at t_i in the process of maximum braking is calculated based on the initial velocity (v_v^0) and deceleration during braking ($a_{v.a}$),

$$v_v(t_i) = v_v^0 + a_{v.a} \cdot t_i \tag{S7}$$

where v_v represent the vehicle velocity. To determine the change of the vehicle velocity and location in the time window, $t_i \,\epsilon\, (0,\ t_v)$ is set as a time sequence with intervals of $\Delta t\ (\Delta t = t_{i+1} - t_i)$, $i = (1, 2, \ldots \ldots, [t_v/\Delta t])$.

In order to maintain the stability of the vehicle body, the minimum turning radius of the vehicle ($R_{v.s}$) depends on the vehicle velocity and the maximum lateral acceleration. Solving the minimum turning radius of vehicle at t_i,

$$R_{v.s}(t_i) = \left(v_v(t_i)\right)^2 / a_{v.al} \tag{S8}$$

Forward moving distance of the vehicle at t_i, $D_v(t_i)$, in the process of maximum braking was calculated based on the initial velocity (v_v^0) and braking deceleration ($a_{v.a}$),

$$D_v(t_i) = v_v^0 \cdot t_i + 0.5 \cdot a_{v.a} \cdot t_i^2 \tag{S9}$$

The steering angle of the vehicle ($A_{v.s}$) at t_i is calculated based on the geometric relationship between vehicle steering radius and moving distance per unit time, i.e., the accumulation of the steering angle in the previous time.

$$A_{v.s}(t_i) = \sum_{i=1}^{[t_i/\Delta t]} tan^{-1}\left(\frac{R_{v.s}(t_i) - \sqrt{{R_{v.s}}^2(t_i) - (D_v(t_{i+1}) - D_v(t_i))^2}}{D_v(t_{i+1}) - D_v(t_i)}\right) \quad \text{(S10)}$$

Moving displacement of vehicle front-end to the right ($D_{v.R}$) at t_v in the process of maximum braking is calculated based on the steering angle and the moving distance per unit time,

$$D_{v.R} = -\sum_{i=1}^{[t_v/\Delta t]}((D_v(t_{i+1}) - D_v(t_i)) \cdot tanA_{v.s}(t_i)) \quad \text{(S11)}$$

where $D_{v.R}$ represent the moving displacement of vehicle front-end to the right.

Determining the hazard distance of pedestrian to centreline of vehicle (equation (S11) and (S2)~(S5)):

$$-\frac{L_{vw}}{2} + D_{v.R} + d_{p.aBA} \leq D_p^0 \leq \frac{L_{vw}}{2} + D_{v.R} + d_{p.aFA} \quad \text{(S12)}$$

Similarly, scenario (I**) denotes the condition that vehicle would be moving at maximum braking and left steering. Moving displacement of vehicle front-end to the left ($D_{v.L}$) at t_v in the process of maximum braking is calculated based on steering angle and moving distance of the vehicle per unit time (eq. (S7)~(S10))

$$D_{v.L} = \sum_{i=1}^{[t_v/\Delta t]}((D_v(t_{i+1}) - D_v(t_i)) \cdot tanA_{v.s}(t_i)) \quad \text{(S13)}$$

Determining the hazard distance of pedestrian to centreline of vehicle (equation (S13) and (S2)~(S5)):

$$-\frac{L_{vw}}{2} + D_{v.L} + d_{p.aBA}(t_v) \leq D_p^0 \leq \frac{L_{vw}}{2} + D_{v.L} + d_{p.aFA}(t_v) \quad \text{(S14)}$$

(II) Pedestrian notice the coming vehicle and would be moving to the "collision venue" at avoiding velocity ($v_{p.a}$); vehicle do not notice the moving pedestrian and would move with the initial- driving velocity.

Solving the time of vehicle to potential collision venue (t_v) based on the vehicle initial velocity (v_v^0) and distance of vehicle arrive at potential collision venue (D_v^0) (Figure 7).

$$D_v^0 = v_v^0 \cdot t_v \quad \text{(S15)}$$

Determining the hazard distance of pedestrian to centreline of vehicle (equation (S15) and (S2)~(S5)):

$$-\frac{L_{vw}}{2} + d_{p.BA}(t_v) \leq D_p^0 \leq \frac{L_{vw}}{2} + d_{p.FA}(t_v) \quad \text{(S16)}$$

(III) Pedestrian do not notice the coming vehicle and would be moving to the "collision venue" with the initial-walking velocity; vehicle notice the moving pedestrian and would move at the maximum braking (no steering included).

Pedestrian would be moving to the "collision venue" at initial-walking velocity. Solving the pedestrian moving distance (d_p).

$$v_{p.a}(t) = v_p^0,\ t \in (0, t_v) \quad \text{(S17)}$$

$$d_p(t_v) = \int_0^{t_v} v_{p.a}(t)dt \quad \text{(S18)}$$

Determining the hazard distance of pedestrian to centreline of vehicle (equation (S1) and (S17)~(S18)):

$$-\frac{L_{vw}}{2} + d_p(t_v) \leq D_p^0 \leq \frac{L_{vw}}{2} + d_p(t_v) \quad \text{(S19)}$$

(IV) Both the pedestrian and vehicle do not notice each other. Pedestrians and vehicle would be moving to the "collision venue" with the initial-velocity.

Determining the hazard distance of pedestrian to centreline (equation (S15) and (S17)~(S18)):

$$-\frac{L_{vw}}{2} + d_p \leq D_p^0 \leq \frac{L_{vw}}{2} + d_p \quad \text{(S20)}$$

III. Movie showing the pedestrian reactions in the experiments

Movie S1. In the movie, we provide animations of the experimental platform and records in the present study to aid the reader in visualizing the pedestrian reactions (Supplementary Figure 1). The video shows the view of the scenario from both the pedestrian and a third-party (i.e., the experimenter), the real in-lab subject motion of the subject, the parallel virtual interaction with the vehicle via VR device, and animations of the kinematic reconstructions of the pedestrian.

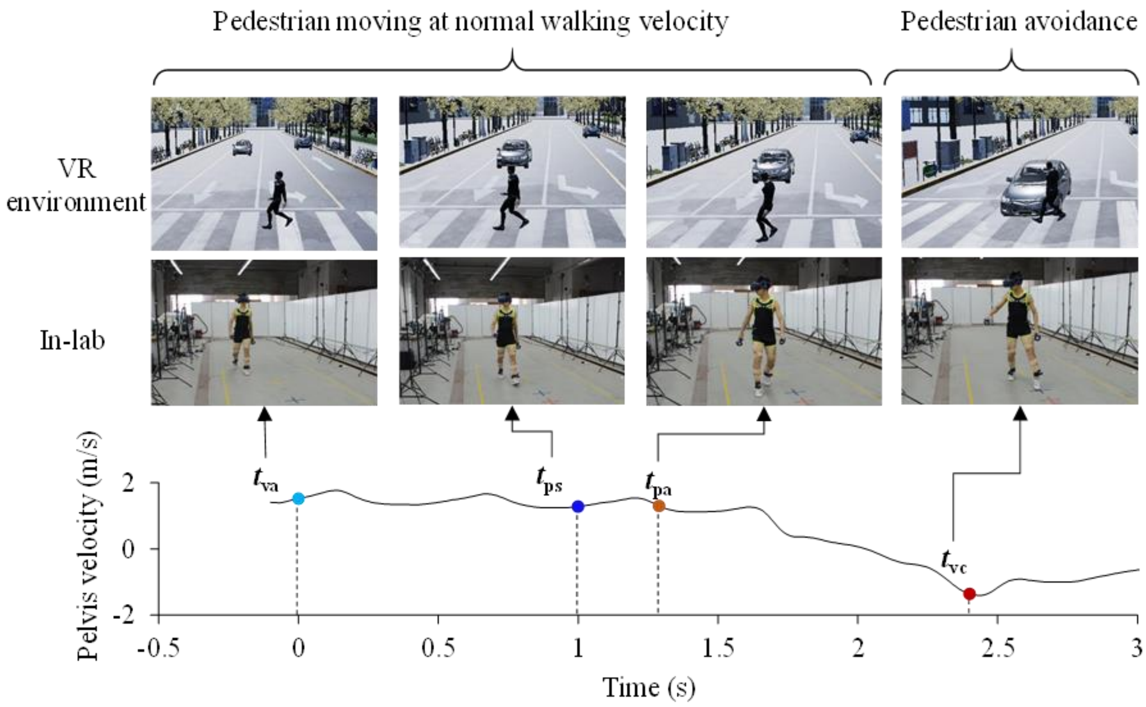

Supplementary Figure 1. Physical and virtual pedestrian reactions in the present experiments (shown as an example: lab experiment photos of Case No. 15).
